# Exploiting the superior protein resistance of polymer brushes to control single cell adhesion and polarisation at the micron scale

**DOI:** 10.1016/j.biomaterials.2010.02.066

**Published:** 2010-06

**Authors:** Julien E. Gautrot, Britta Trappmann, Fabian Oceguera-Yanez, John Connelly, Ximin He, Fiona M. Watt, Wilhelm T.S. Huck

**Affiliations:** a Melville Laboratory for Polymer Synthesis, Department of Chemistry, University of Cambridge, Lensfield Road, Cambridge CB2 1EW, UK; b Wellcome Trust Centre for Stem Cell Research, University of Cambridge, Tennis Court Road, Cambridge CB2 1RE, UK; c Radboud University Nijmegen, Institute for Molecules and Materials, Heyendaalseweg 135, 6525 AJ Nijmegen, The Netherlands

**Keywords:** Polymer brush, Extra-cellular matrix, Patterning, Single cell, Cell polarisation

## Abstract

The control of the cell microenvironment on model patterned substrates allows the systematic study of cell biology in well defined conditions, potentially using automated systems. The extreme protein resistance of poly(oligo(ethylene glycol methacrylate)) (POEGMA) brushes is exploited to achieve high fidelity patterning of single cells. These coatings can be patterned by soft lithography on large areas (a microscope slide) and scale (substrates were typically prepared in batches of 200). The present protocol relies on the adsorption of extra-cellular matrix (ECM) proteins on unprotected areas using simple incubation and washing steps. The stability of POEGMA brushes, as examined via ellipsometry and SPR, is found to be excellent, both during storage and cell culture. The impact of substrate treatment, brush thickness and incubation protocol on ECM deposition, both for ultra-thin gold and glass substrates, is investigated via fluorescence microscopy and AFM. Optimised conditions result in high quality ECM patterns at the micron scale, even on glass substrates, that are suitable for controlling cell spreading and polarisation. These patterns are compatible with state-of-the-art technologies (fluorescence microscopy, FRET) used for live cell imaging. This technology, combined with single cell analysis methods, provides a platform for exploring the mechanisms that regulate cell behaviour.

## 1 Introduction

Cell behaviour is regulated by extrinsic environmental cues, as well as soluble cytokines and growth factors. In addition, binding of extra-cellular matrix (ECM) proteins and cell-membrane ligands are modulated by physical parameters such as matrix stiffness and ligand spatial distribution (topology and topography) [1,2]. Studying how cells respond to these signals is of great importance for fully understanding tissue homeostasis and the development of diseases such as cancer. However, it is not always possible to probe these interactions in their *in vivo* context and therefore *in vitro* approaches are required [3]. In order to recreate and model some of the cues defining the cell microenvironment *in vitro*, matrix engineering techniques have been developed to model cell-ECM or cell-cell interactions and integrate physical parameters (stiffness, topology, topography) [4].

Micro-patterning, which allows the control of cell spreading in well defined areas, is particularly useful for both mimicking the cell microenvironment [5,6] and developing screens based on cell arrays [7,8]. For example, manipulating the shape and size of cell clusters allows the control of differentiation of embryonic stem cells [9], of the branching of mouse mammary epithelial tubules [10] and induces the polarisation of primary rat astrocytes [11]. At the single cell level, spreading and cell shape can be controlled via the design of ECM protein patterns at the micron scale (typically 5–100 μm). In the case of circular adhesive islands, varying the size of the pattern modulates cell behaviour via changes in cell shape and their effect on the cytoskeleton and the mechano-transduction machinery [12,13]. Lowering the degree of symmetry of the adhesive pattern (e.g. by using squares, ellipses, triangles or tear shapes) results in an equal decrease in the cell symmetry and correlates with the orientation of the cell division axis [14], the direction of cell migration [15] and more generally cell polarisation [16].

The generation of ECM protein patterns on the micron-scale that are suitable for keeping a tight control over cell spreading for extended periods of time has necessitated the development of new techniques, mainly based on photo-lithography and micro-contact printing (μCP). These must meet several key requirements: (1) the resolution of the pattern should be excellent down to one micron on sufficiently large substrate areas so that a meaningful number of cells can be studied; (2) the background defining the ECM pattern should be highly resistant to both protein and cell adsorption for extended periods of time (>24 h), in order to provide tight control over cell spreading; (3) the ECM protein deposited should adhere strongly to the patterned shape, in order to resist the pull exerted by cells upon spreading (which can result in cell rounding and detachment); (4) the patterning protocol used should be simple and transposable to biology labs, ideally consisting of series of incubations and washes; (5) the final substrate should be compatible with high resolution imaging techniques. Despite the wealth of novel approaches for single cell patterning, a combination of these requirements remains elusive. In addition, reports in which these different aspects are investigated in parallel are scarce.

Polymer brushes are a relatively novel type of coating used for modifying the surface properties of materials [17,18] and display key properties for high fidelity single cell patterning. In particular poly(oligo(ethylene glycol methacrylates)) display excellent protein resistance (even in the presence of complex protein mixtures such as sera and blood plasma [19,20]), resist cell adhesion unless functionalised with cell-membrane ligands [21,22] and can be patterned on the micron scale via simple micro-contact printing [23]. In addition, derivatives bearing lateral hydroxyl groups can be further functionalised with biomolecules without significantly impairing the protein resistance of the coating [24,25], thereby enabling the generation of binary biofunctional patterns.

In the present work, we show that poly(oligo(ethylene glycol methyl ether methacrylate)) (POEGMA) enables the formation of high fidelity ECM protein patterns that control the spreading of single cells. The synthesis and chemical stability of POEGMA brushes is first investigated using a combination of ellipsometry and surface plasmon resonance (SPR). The key parameters controlling the formation of high quality ECM patterns are then studied via immuno-fluorescence labelling and atomic force microscopy (AFM), both on ultra-thin (2–15 nm) gold deposited on glass and on uncoated glass substrates. The spreading of cells on the resulting patterns and the induction of polarisation using asymmetric islands demonstrate the potential of this technique for controlling the behaviour of single cells. Finally, the compatibility of these patterned substrates with state-of-the-art imaging technologies is established.

## 2 Materials and methods

### 2.1 Materials for patterning

Oligo(ethylene glycol methyl ether methacrylate) (OEGMA, Mw 300), CuCl, CuBr_2_, 2,2′-dipyridyl (bpy), 3-aminopropyltrimethoxysilane (APTS, 97%, Sigma), poly(sodium 4-styrene sulfonate) (PSS, Mw 70,000, Sigma), poly(allylamine hydrochloride) (PAA, M_w_ 56,000 Sigma), bovine serum albumin (BSA, ≥96%, cell culture tested grade) and phosphate-buffered saline (PBS, 150 mm) were purchased from Aldrich and used as received. ω-Mercaptoundecylbromoisobutyrate (**1**) [26], silane (**2**) [27] and macro-initiator (**3**) [28] were synthesised according to the literature. Deionised water was obtained using a Synergy system from Millipore.

### 2.2 Materials for cell culture and immuno-staining

Collagen (rat, type I, BD Biosciences, San Jose, CA), FITC-conjugated collagen I (from bovine skin, Sigma), fibronectin (from bovine plasma, Sigma), laminin (from human placenta, Sigma), bovine serum albumin (>96%, Sigma), sterile PBS (PAA Laboratories), trypsin (0.25%, Gibco), versene (Gibco), keratinocyte serum-free medium (KSFM, Gibco, Carlsbad, CA), foetal bovine serum (FBS, PAA Laboratories), bovine serum (BS, PAA Laboratories), horse serum (HS, PAA Laboratories), were used as received. Complete FAD medium was prepared as follows: 1 part Ham's F12, 3 parts DMEM, 10% FBS, 0.5 μg/ml hydrocortisone, 5 μg/ml insulin, 10^−10^ m cholera toxin, 10 ng/ml EGF. Antibodies raised against collagen I (mouse monoclonal, COL-1, Sigma), vinculin (mouse monoclonal, Hvin1, Sigma) and mouse IgG (H + L, Alexa Fluor 488 and 594 conjugates, raised in Donkey and Goat, respectively, Invitrogen) were used at the recommended dilutions. TRITC-phalloidin reconstituted in methanol was from Sigma Aldrich (St. Louis, MO). DAPI for nuclear staining was from Sigma.

### 2.3 Substrate preparation

Gold-coated substrates were prepared via evaporation of a chromium layer followed by evaporation of gold on 0.13 mm glass slides (thickness no 1, borosilicate glass, freshly plasma oxidized using air plasma, K1050X from Emitech), using an Edwards Auto 500 evaporator. Three different types of gold substrates were prepared, with increasing gold thicknesses: 2, 4 and 15 nm (these thicknesses refer to the thickness of gold (Birmingham Metals) deposited on the glass slides as evaluated using a quartz crystal microbalance). The corresponding thicknesses of chromium were 0.5, 0.5 and 1.5 nm, respectively. Substrates coated with 15 nm gold were used throughout the study and referred to as “gold substrates” except in the section dealing with live imaging, where all three thicknesses were used.

Glass substrates for patterning using silane **2** or macro-initiator **3** were prepared using 0.13 mm glass slides (freshly oxidized with plasma) and used directly for micro-contact printing or after incubation in an APTS ethanolic solution (overnight, 5 mm). For micro-contact printing with macro-initiator **3**, APTS-coated slides were then incubated sequentially in aqueous solutions of PSS (1 mg/mL in deionised water, pH 1) and PAA (1 mg/mL in deionised water, pH1). This process was repeated twice, as described in the literature [28].

### 2.4 Master preparation

SU8-2005 and SU8-2025 photoresists (MicroChem) were spin-coated on Si wafer to a final film thickness of 5 μm and 25 μm respectively, as measured by profilometry on the final master (DekTak 150). After spin-coating, the wafer was prebaked (at 65 °C for 1 min, then at 95 °C for 2 min and finally at 65 °C for 1 min), and then exposed to UV light through a photomask (Circuitgraphics) on a mask aligner (MJB4, Suss MicroTec). After postbaking at 65 °C for 1 min and then at 95 °C for 2 min, the master was developed for 3 min, followed by hard baking at 170 °C for 15–40 s.

### 2.5 Micro-contact printing

For micro-contact printing on gold substrates, a 5 mm ethanolic solution of **1** was spread on a PDMS (184 silicone elastomer, Sylgard) stamp using a cotton bud for 10 s. The stamp was washed thoroughly using ethanol, dried in a stream of nitrogen and deposited on the gold substrate, allowing conformal contact to occur for 10 s. The substrates were then kept in a desiccator under vacuum until all were ready for polymerisation.

For micro-contact printing of silane **2** on glass or APTS-coated glass, a solution of **2** in dry hexane (1 μl in 3 mL, puriss grade over molecular sieves, Fluka) was inked using a cotton bud onto a flat PDMS stamp (generated against air) for 10 s. The stamp was dried in a stream of nitrogen for 15 s and placed in conformal contact with the moulded PDMS stamp (using masters prepared as detailed above) for 45 s. The moulded stamp was placed in conformal contact with the glass substrate for 25 s. The substrates were then kept in a desiccator under vacuum until all were ready for polymerisation.

For micro-contact printing of macro-initiator **3** on layer-by-layer-coated glass, a PDMS stamp was oxidized in plasma for 15 s, inked with a solution of macro-initiator (1 mg/mL in deionised water, pH 1) for 15 min and spinned at 3000 rpm for 30 s (Delta 10TT spin coater from Suss MicroTec) to remove the excess initiator solution. The resulting stamp was placed in conformal contact with the glass substrate for 5 min. The substrates were then kept in a desiccator under vacuum until all were ready for polymerisation.

### 2.6 Polymer brush growth

For the growth of a 20 nm-thick POEGMA brush: a solution of CuBr_2_ (9 mg, 40 μmol), bpy (160 mg, 1.0 mmol) and OEGMA (6.3 g, 17.5 mmol) in water/ethanol 4/1 (15 mL) was degassed using nitrogen bubbling for 30 min. CuCl (41 mg, 410 μmol) were added to this solution and the resulting mixture further degassed for 15 min before transferring to a flask containing the initiator-coated gold surface under inert atmosphere. The polymerisation was stopped after 15 min by immersing the coated substrates in deionised water, followed by washing with copious amounts of ethanol and drying in a nitrogen stream.

### 2.7 ECM protein deposition

The POEGMA-patterned substrates (cut into 1 cm^2^ chips) were placed in a 24-well plate (tissue culture treated, polystyrene, Corning) and immersed in 70% ethanol for 10 min before aspirating and washing with PBS twice. A solution of ECM protein (collagen I, fibronectin or laminin at 20, 10 and 10 μg/mL, respectively – 0.5 mL/well) was poured onto the substrate and the plate was left to incubate at 37 °C for 45–60 min. A dilute HCl solution (1 mm) was added to each well, the solution aspirated without allowing the substrates to be exposed to air, and the procedure repeated twice. The solution was then aspirated to dryness and the substrates were washed once more with dilute HCl and twice with PBS. Alternatively, PBS can be used instead of HCl, without significantly impairing the quality of the patterns.

### 2.8 Cell culture and seeding onto patterned substrates

Primary human epidermal keratinocytes (HEKs) isolated from neonatal foreskin were maintained with feeder J2 3T3 fibroblasts as previously described [29]. The feeder cells were removed using a versene solution (no trypsin), and HEKs (passage 2–8) were trypsinized (versene/trypsin 4/1 from stock solutions) and re-seeded onto the micro-patterned substrates (coated with the relevant ECM protein, in a 24-well plate) at a density of 25,000/cm^2^ (50,000 cells/mL, 0.5 mL/well) in KSFM or FAD medium. Cells were allowed to adhere for 1 h before rinsing three times with fresh medium (0.5 mL each time). Care must be taken not to let the substrate dry during the washing step.

NIH-3T3 cells were maintained in DMEM (Invitrogen) containing 10% FBS. Transfection of cDNA into the cells was performed with the Lipofectamine™ 2000 transfection reagent (Invitrogen) using Opti-MEM as per the manufacturer's instructions. The day after transfection the cells were harvested and recovered using a MoFlo flow cytometer (Dako). A threshold of autofluorescent cells was set with non-transfected cells and the cells expressing C5V were collected on the basis of their fluorescence intensities ranging from 10^1^ to 10^2^. Dead cells were excluded by DAPI staining. The C5V and CTV FRET standards constructs were the kind gift of Steven S. Vogel, NIH, Bethesda, MD, USA.

### 2.9 Immuno-staining and microscopy

Patterned cells were fixed and permeabilised simultaneously in 4% PFA and 0.2% Triton-X100, blocked for 1 h in 10% FBS containing 0.25% gelatin, incubated at room temperature with primary antibodies for 1 h, incubated with conjugated secondary antibodies for 1 h and mounted on glass slides in Mowiol. TRITC-phalloidin and DAPI were included in the secondary antibody solution. Images were acquired with a Leica DMI4000 fluorescence microscope (CTR 6000 laser, excitation filter BP480/40, suppression filter B527/30, dichromatic mirror 505).

### 2.10 Image and statistical analysis

Images were analysed using ImageJ. For quantifying cell spreading, images were thresholded using the level of the background as lower threshold and a “particle analysis” was performed on the resulting binary images. For generating overlays of stainings for collagen I, F-actin or vinculin, images were aligned using the FITC-collagen I channel (staining for the adhesive pattern) and centered. A stack of images was generated and the overlay image was obtained using the z-projection of the stack. The MAX option was used for vinculin stainings, in order to reduce the importance of the background, and the SUM option was used for all other stainings.

Single cell spreading data were analyzed via one-factor ANOVA and Tukey's test for posthoc analysis. Significance was determined by *P* < 0.01.

### 2.11 Live imaging

For long-term (1 week) live imaging, substrates were mounted in a 24-well plate (ImageLock, Essen Instruments) using PDMS as glue and allowed to set for 24 h. Cells were seeded as described above, in KSFM. Culture medium was changed every 2–3 days. Images of the same spots were taken using an IncuCyte from Essen Instruments.

Live confocal fluorescence and FRET microscopy of NIH-3T3 fibroblasts (expressing C5V or either the single donor Cerulean or the acceptor Venus) was carried out using a Leica TCS-SP5 confocal microscope equipped with a 63 × 1.4–0.6NA, Oil, HCX Plan-Apo blue corrected objective lenses. A variant of ECFP, Cerulean was excited with an argon laser at 458 nm and the detector band was set at 470–500 nm. The variant of EYFP, Venus was excited with the argon laser at 514 nm and the detector band was set at 530–625 nm. The exposure times were kept equal for all the images and selected for their intensities to be within the linear range of the detectors. Images of single cells expressing C5V, Cerulean or Venus were acquired in order to calculate the donor spectral bleed-through (DSBT), the acceptor spectral bleed-through (ASBT) and the corrected FRET using the PFRET algorithm kindly provided by Ammasi Periasamy (W.M. Keck Center for Cellular Imaging, Charlottesville, VA, USA) [30].

### 2.12 Instrumental analyses

Ellipsometry measurements were performed with a α-SE instrument from J.A. Woolam at 70^o^ incidence angle. A simple gold substrate/cauchy film model was used and fitted between 400 and 900 nm.

Surface plasmon resonance (SPR) was performed on a Biacore 3000. SPR chips (Ssens) were coated with a POEGMA brush (20–30 nm thick, see polymerisation procedure above), dextran (Akubio) or a tri(ethylene glycol) **4** monolayer (triethylene glycol mono-11-mercaptoundecylether, Sigma, deposited from a 5 mm ethanolic solution overnight). Mounted chips were docked, primed with buffer (PBS) twice and equilibrated at 20 μL/min for 30 min or until a stable baseline was obtained. The chips were exposed to the protein or serum of interest for 5 min and washed with PBS for 10 min. The amount of non-specifically bound mass was measured after 10 min washing. The flow rate was 10 μL/min. Measurements were carried out in triplicate.

AFM images were obtained using a Dimension 3100 equipment from Digital Instruments (Santa Barbara, CA) in tapping mode. UV–vis absorption spectra of gold-coated substrates were acquired on a Cary 4000 photospectrometer from Varian.

## 3 Results and discussion

### 3.1 Brush growth, stability and protein resistance

The growth of POEGMA brushes using atom transfer radical polymerisation (ATRP) conditions is particularly well-controlled, as reported in the present study and the literature ([19,20] and Fig. S1). The commercially available oligo(ethylene glycol) methacrylate monomers, with various side chain lengths and terminal chemical groups, that have been studied to date display controlled growth even to thick brushes (200–300 nm), using classical ATRP catalytic systems (Cu(I)Clbpy_2_ complex) and aqueous conditions. In addition, the grafting density of these coatings can be manipulated easily, resulting in a range of physical, morphological and swelling properties ([18,26] and Fig. S1). Several methods can be used to control the rate of the brush growth: varying the monomer concentration, the Cu(I) to Cu(II) ratio, the nature of the ligands and the solvent composition (addition of alcohols such as methanol or ethanol) ([18] and Fig. S1). The method that was preferred was the use of ethanol, as it results in excellent control over the polymerisation especially for thin brushes such as those used in the present work (the thickness profile is linear with no initial jump).

The stability of POEGMA brushes was found to be excellent in ambient conditions. The degradation and cleavage of polymer brushes may result in significant impairment of surface properties such as rheological behavior and protein resistance. Klok and co-workers reported the detachment of POEGMA brushes from glass [31]. In their study, sheets of brush detached, as evidenced by clear images of folded films remaining partly attached to the substrate. We did not observe brush detachment either on gold, silicon or glass substrates. This difference may be due to the fact that Klok and co-workers used hydroxyl-terminated monomers (commercially available from Sigma), which are known to contain significant amounts of di-methacrylates ([32] and mass spectrograms of the commercial compounds), therefore leading to cross-linked coatings which may detach as sheets rather than free polymer chains. In addition, the brushes used in the present study are relatively thin, which may reduce the entropic pressure imposed by chain stretching, as was proposed by Klok and co-workers [31]. Long-term (up to two months) stability studies conducted in PBS at room temperature clearly showed that brush cleavage was minimal (Fig. S2). Both the brush thickness (up to 150 nm) and density (down to 5% starting initiator molecules) were found to have little effect and the maximal reduction in thickness remained within 10%. This excellent stability suggested that any decrease of the protein resistance properties of POEGMA brushes would originate from chemical degradation rather than decrease in the thickness and therefore the physical barrier to protein diffusion and adsorption to the underlying substrate.

**Fig. 1:**
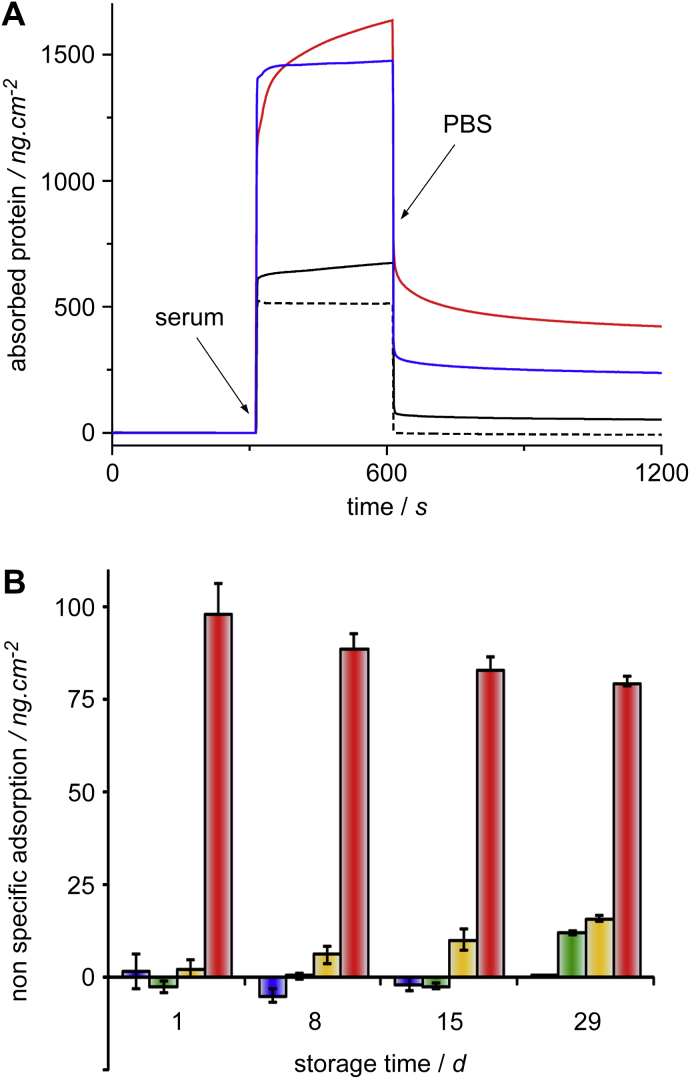
Protein resistance of POEGMA brushes. (A) Exposure of coatings to sera, monitored by SPR; dextran (red line), triethylene glycol **4** SAM (blue line) and a POEGMA brush (30 nm, black line) were exposed to whole horse serum for 5 min; a POEGMA brush (30 nm, black dashed line) was exposed to foetal bovine serum for 5 min. (B) Stability of POEGMA brushes upon storage: brushes were stored in ambient conditions (bench drawer) and their protein resistance to foetal bovine (blue, 10%, green, 100%) and bovine (orange 10%, red, 100%) sera was measured by SPR (exposure for 5 min, followed by 10 min washing with PBS, at a flow rate of 10 μl/min). Measurements were carried out in triplicates.

The protein resistance of POEGMA brushes was found to be significantly improved compared to other frequently used coatings such as dextran and tri(ethylene glycol) **4** monolayers used in immobilization platforms or for cell patterning (Fig. 1). The protein resistance of POEGMA brushes, in some cases even after chemical modification, was found to be excellent and falls only short of the performance of another type of polymer brushes, based on zwitterionic monomers [33]. In order to measure the protein resistance of such ultra-low fouling coatings, single protein solutions are not sufficient as, even at high concentrations, only low levels of protein density are measured, which are difficult to quantify reliably (especially using label-free detection methods such as quartz crystal micro-balance or SPR) [33]. Therefore, complex protein mixtures such as blood plasma or serum are better suited and more relevant to applications such as medical diagnostics and cell patterning, which typically require the exposure of surfaces to these media. We found that, when exposed to whole horse serum, the level of non-specifically bound mass measured for POEGMA brushes was 4- and 8-fold lower than those of tri(ethylene glycol) **4** monolayers and dextran, respectively (Fig. 1A). In order to estimate the effect of storage and eventual chemical degradation on protein resistance, we stored POEGMA brush coated SPR chips in ambient conditions (a bench drawer) for a period of one month and measured the level of non-specifically bound mass when exposed to bovine and foetal bovine sera (BS and FBS both undiluted and at 10% concentration) (Fig. 1B). These sera were chosen because of their relevance to cell culture conditions, which often make use of media containing no serum or 10% BS or FBS. The non-specific binding measured for FBS was extremely low (initially below 2 ng/cm^2^) and only increased moderately for undiluted serum after one month of storage. Similarly, in the case of diluted BS, the non-specific adsorption increased gradually to moderate levels (16 ng/cm^2^) over the same period (whereas adsorption from undiluted BS remained high, between 75 and 100 ng/cm^2^). Hence, when exposed to media typical of tissue culture conditions (10% sera), POEGMA brushes retained excellent protein resistance properties, even after one month of storage.

### 3.2 ECM protein deposition onto gold substrates

**Fig. 2:**
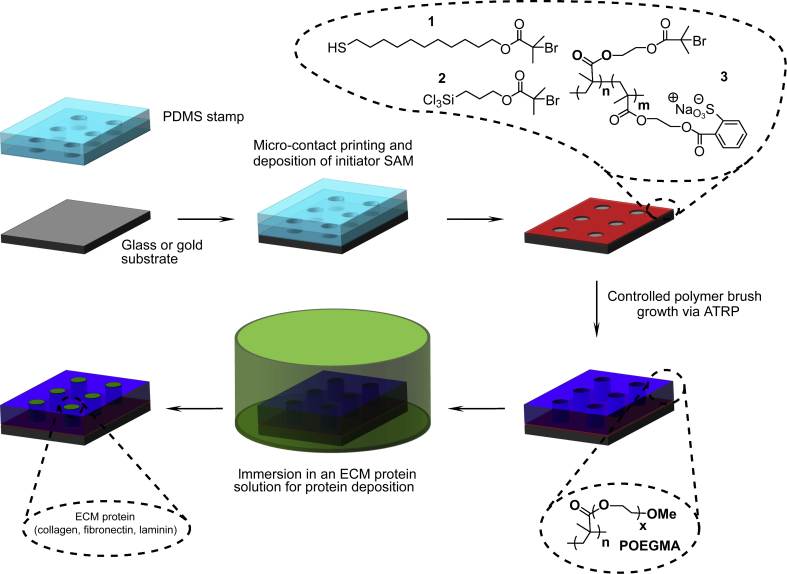
ECM protein patterning protocol. An initiator molecule or macromolecule is micro-contact printed on a substrate (gold-coated glass or glass), followed by POEGMA brush growth. At this stage, substrates can be stored in ambient conditions for several weeks without impairing their properties. For ECM protein deposition, a substrate is immersed in a solution of the desired protein, washed and used for cell seeding or immuno-staining.

**Fig. 3:**
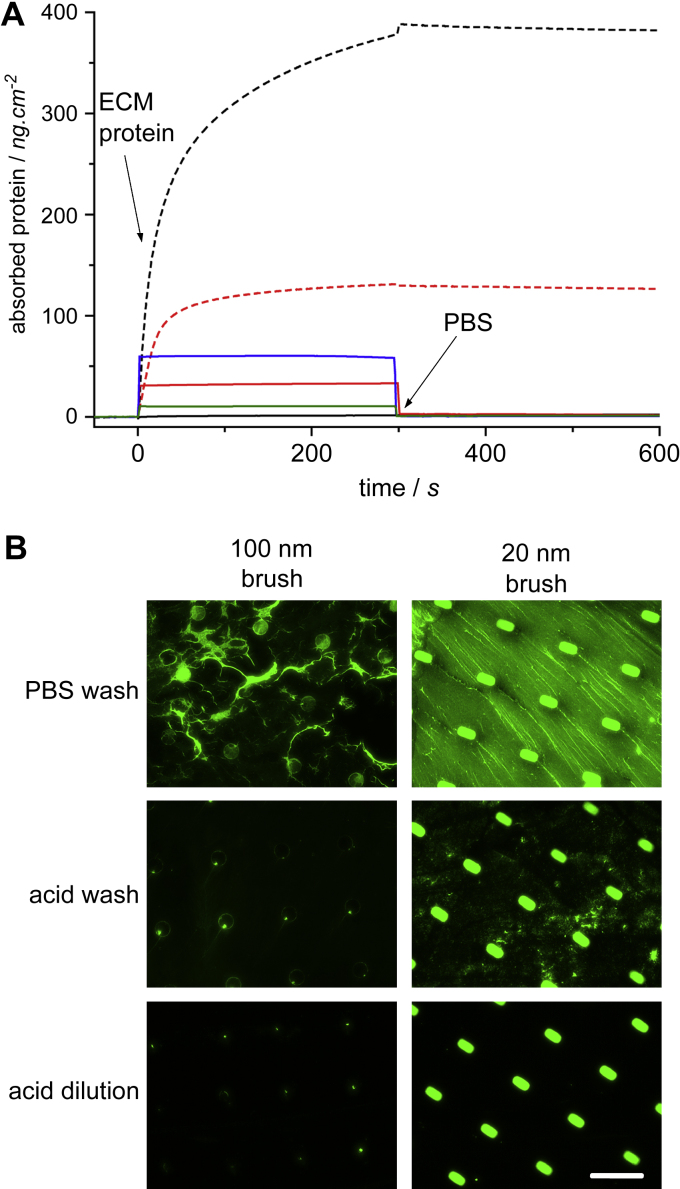
ECM protein deposition on gold islands. A, SPR traces displaying the exposure of POEGMA brushes (solid lines) and bare gold chips (dashed lines) to ECM proteins (black, collagen I; red, fibronectin; blue, laminin; green, BSA). B, Deposition of collagen I on substrates patterned with POEGMA brushes; two different brush thicknesses are compared and three different washing protocols (washing with PBS or dilute acid and dilution of the collagen solution with dilute acid prior to washing with PBS); the shapes of the patterns used for 20 and 100 nm brushes are different; scale bar: 100 μm.

The growth of polymer brushes from self-assembled initiator monolayers is well suited to soft lithography. We developed a simple protein patterning protocol based on the deposition of an initiator monolayer (thiol for gold and silane for glass or silicon) using micro-contact printing and the adsorption of a protein of interest from solution (Fig. 2). Therefore, this protocol does not require the direct micro-contact printing of a particular protein, which can be sometimes unreliable, requires optimisation when the nature of the protein is changed and involves steps during which the protein deposited is dried, which may result in the loss of activity or binding ability for some proteins [34]. This simple procedure is enabled by the excellent protein resistance of POEGMA brushes: exposure of a bare gold surface to ECM protein solutions such as collagen I and fibronectin, results in high protein densities (100–400 ng/cm^2^) (Fig. 3A). In comparison, exposure of POEGMA to the same protein solutions gives rise to SPR signals indistinguishable from the noise. Hence, POEGMA can be used as a background delimiting an unprotected area onto which a protein can be deposited directly from solution, by simple differential adsorption. The quality and lateral resolution of the protein pattern obtained is determined by that of micro-contact printing and the quality of the mask and master used for generating the PDMS stamp (routinely down to 5–10 and 1–2 microns for acetate and chromium masks, respectively).

In order to obtain high quality cell-adhesive patterns, the protocol used for ECM protein deposition was found to be critical. In particular, collagen is a difficult protein to pattern because of its tendency to form gels and fibres at neutral pH and moderate concentrations [35]. If the collagen solution was simply removed from the incubation-well containing the substrate before washing with a PBS solution, immuno-fluorescence staining showed that collagen fibres deposited randomly on the surface, although an intense staining was also observed on the unprotected island (Fig. 3B). This suggested that deposition on the bare substrate was occurring but that some ECM protein also adhered to POEGMA brushes, in strong contrast with SPR results (Fig. 3A). We hypothesised that this discrepancy arose from the removal of the ECM solution before the washing step, which does not occur in SPR protocols and allows the POEGMA surface to dry and trap infiltrated proteins. The relative hydrophobicity of POEGMA brushes is well documented [22,36] and Chilkoti and co-workers reported that it was possible to irreversibly immobilise antibodies using these coatings by allowing a drop of protein solution to dry on their surface [37]. When the collagen solution was diluted with a weak acid solution just after incubation, the background positive staining disappeared, as predicted by SPR results (Fig. 3B). Similarly, dilution with PBS resulted in clean ECM patterns, although a faint staining was sometimes seen on the background (results not shown). Therefore if a protein is sensitive to dilute acids, it is possible to deposit it using a PBS dilution step. Simple washing with an acid solution after removal of the protein solution decreased to some extent the background staining but did not prevent it (Fig. 3B).

The thickness of the brush was found to be critical for obtaining high densities of ECM protein on the island. When thick (100 nm) brushes were used for the background, the staining obtained on the island was much fainter than for thin (20 nm) brushes (Fig. 3B). This observation suggests the presence of some POEGMA brushes on the island. These may be due to lateral diffusion of the initiator ink during micro-contact printing [38–40]. AFM images gave some evidence of such lateral diffusion phenomenon, as the profile of thick brushes is slightly rounded in comparison to that of thin brushes (Fig. S3). When the time of polymerisation is increased in order to obtain thick brushes, the initiator molecules that have diffused onto the island can generate a sufficiently thick layer preventing protein adsorption.

The adsorption of ECM proteins directly from solutions easily enables the deposition of proteins blends with well defined compositions. For some applications, it may be useful to coat adhesive islands with more than one (ECM) protein. For example, by using solutions containing collagen I and BSA in a range of relative concentrations, it is possible to control the density of ECM protein deposited on the surface (as estimated by immuno-fluorescence staining, see Fig. S4). The use of a mixed deposition approach is an advantage compared to depositions of a single protein at different concentrations as the dynamic range may be expected to be wider and more reproducible (as the total amount of protein in solution remains constant).

### 3.3 Patterning of glass substrates

Glass patterning of ECM proteins is important because live imaging of cells seeded onto such patterns is expected to be of superior quality. Polymer brushes can be grown on glass, silica and silicon substrates, typically using silane initiators [18]. We used three different protocols for the micro-contact printing of an initiator molecule onto glass and investigated their impact on the deposition of collagen: the first two are based on the use of a trichlorosilane **2** for printing via transfer with an intermediate PDMS stamp (see Section 2) onto bare glass or APTS-coated glass; alternatively macro-initiator **3** was printed on a glass coated with a layer-by-layer (LBL) polyelectrolyte assembly. The use of an APTS-coated glass surface was intended to favour the reaction of the silane with the substrate and slow down its lateral diffusion. The macro-initiator **3** was expected to diffuse very little due to its large size and binding to the substrate via electrostatic interactions (rather than via a reactive chlorosilane which, in addition, may hydrolyse during the stamping process).

**Fig. 4:**
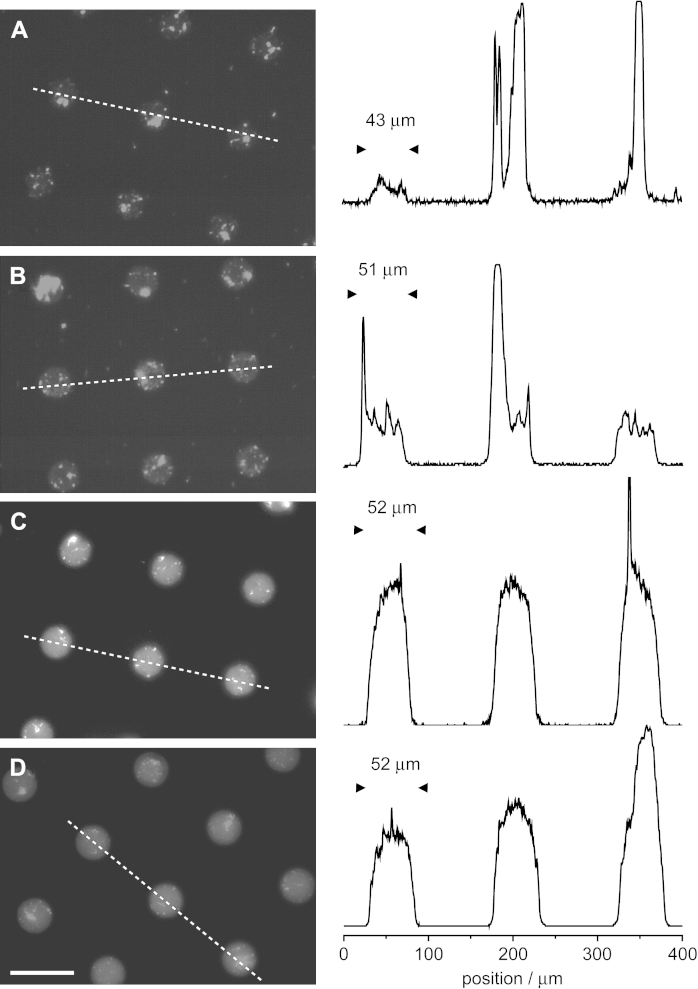
Deposition of collagen I on patterns generated on various substrates. POEGMA brushes were patterned on glass (A), APTS-coated glass (B), LBL-coated glass (C) and gold and substrates were subsequently incubated in collagen I solutions, immuno-stained and imaged via fluorescence microscopy. Height profiles were taken along the dashed lines. Scale bar: 100 μm.

Lateral diffusion over the surface as well as diffusion through the vapour phase of the initiator during printing was found to considerably alter the collagen deposition and final density on the island as well as the control of the feature size. Direct patterning of glass with silane **2** was sensitive to the stamping time: when the conformal contact was maintained for 25 s the transversal profile of the brush imaged by AFM was less rounded than for 45 s stamping (time which allows more initiator molecules to diffuse onto the islands – Fig. S3). Coating the glass substrate with APTS prior to stamping improved the sharpness of the feature and, similarly, the collagen I intensity profile (Fig. 4). Sharper transitions between the collagen density on the island and the background were observed and the diameter of the collagen coated areas was well matched to that of the printed feature (43 μm for bare glass substrate and 51 μm for APTS-coated glass for an expected 50 μm island, Fig. 4). Optimal collagen patterns were obtained for substrates printed using macro-initiator **3**. The use of a macromolecular initiator further reduced lateral diffusion, resulting in sharp AFM profiles, and the underlying polyelectrolyte multilayer improves the adsorption of collagen onto the uncoated islands. Indeed the use of a polyelectrolyte multilayer both allows the facile deposition of the initiator and strengthens protein adsorption.

### 3.4 Cell patterning, spreading and control of polarisation

**Fig. 5:**
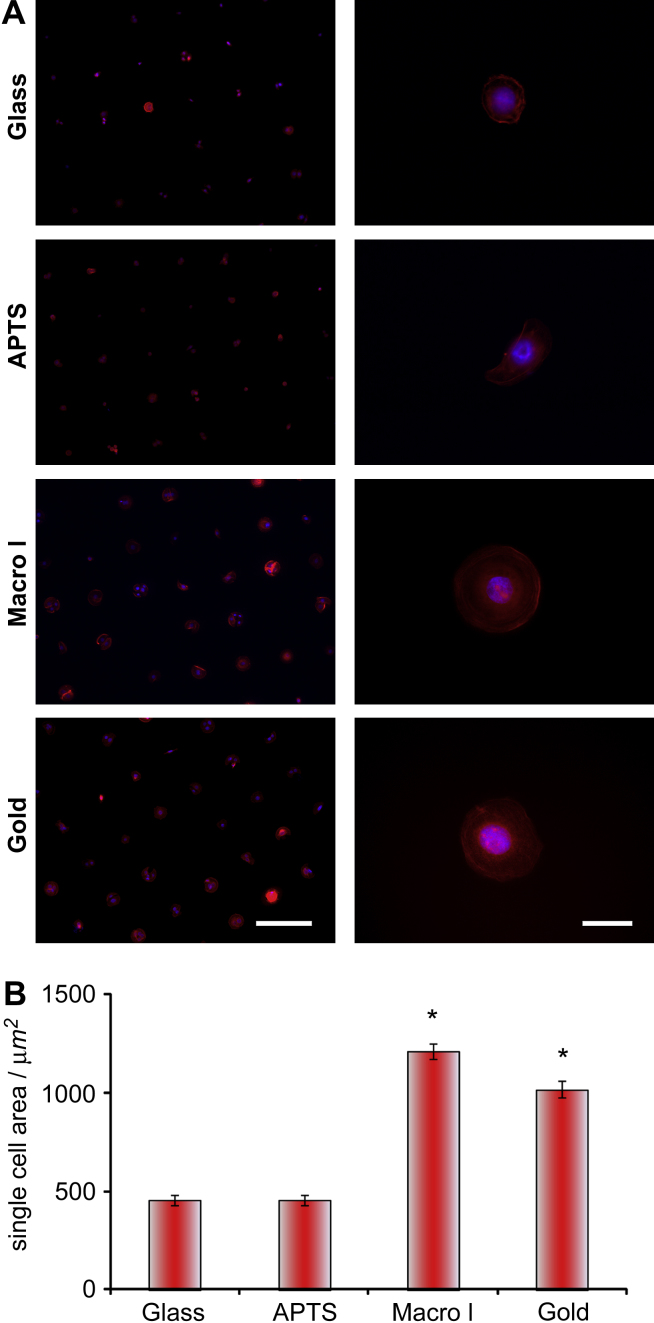
Patterning and spreading of HEKs on POEGMA-patterned substrates. A, Images of cell arrays (left, scale bar: 200 μm) and single cells (right, scale bar: 30 μm) spreading on 50 μm islands, 3 h after seeding (staining: red, F-actin, blue, DAPI); the substrates used were untreated glass (Glass), APTS-coated glass (APTS), LBL-coated glass with macro-initiator printing (Macro I) and gold (Gold). B, Projected cell areas (single cells only) measured from F-actin staining images (at least 100 cells for each substrate); mean ± SD; *P < 0.01.

**Fig. 6:**
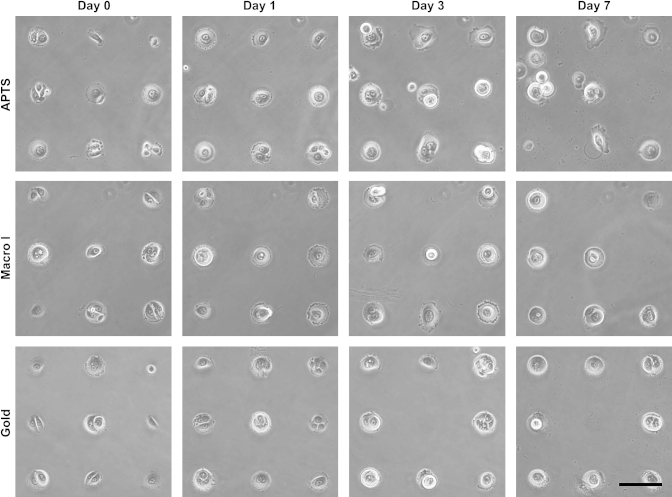
Evolution of HEK arrays. HEKs were seeded on POEGMA patterns generated on APTS-coated glass (APTS), LBL-coated glass with macro-initiator printing (Macro I) and gold (Gold) substrates and incubated for 7 days. For each substrate, the same areas of interest were followed at 0, 1, 3 and 7 days. Scale bar: 100 μm.

Having demonstrated the ease with which both gold and glass substrates may be patterned with ECM proteins using a brush-based background, the patterning of primary human epidermal keratinocytes was studied. HEKs were seeded on collagen I-coated islands (50 μm in diameter). After 1 h the substrates were washed to remove non-adhered cells and examined 3 h after initial cell-plating. On all four types of substrates (gold, glass, APTS-coated glass and macro-initiator patterned glass), HEKs were patterned on relatively large areas of the substrates (Fig. 5), regardless of whether seeding was carried out in the presence or absence of serum. Seeding in the absence of serum on islands that had not been coated with ECM proteins resulted in no cell adhesion, whether on gold or glass substrates. However after 3 h, the spreading of cells was substantially higher on glass patterned with macro-initiator **3** and gold than on the two other types of substrates. This behaviour may be due to the higher density of collagen on islands of the gold and macro-initiator patterns, or to the stronger adsorption of collagen (cells may be able to pull stronger on the ECM molecules without significant surface desorption). Cells remained patterned on 50 μm islands for a period of several days (monitored up to 1 week) without significant loss in the quality of the pattern, except for those generated by micro-contact printing on APTS-coated glass, which started crawling out of the adhesive areas after 3 days (Fig. 6). Therefore the protein resistance of POEGMA-brushes is maintained in cell-culture conditions for long periods of incubation.

**Fig. 7:**
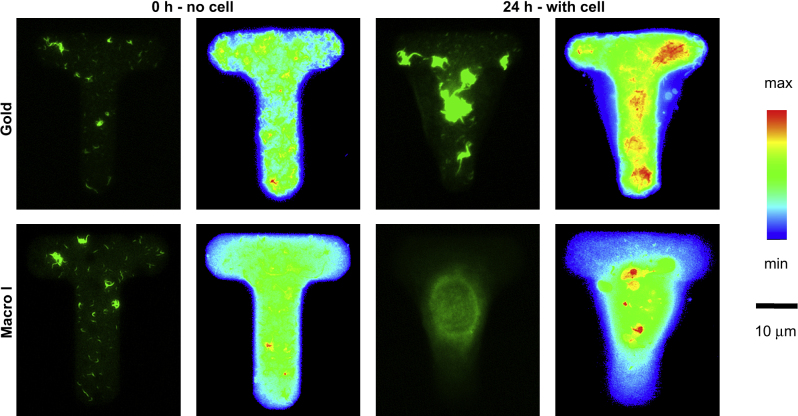
Collagen redistribution by single HEK cells. HEKs were seeded on [T]-shaped collagen I-FITC islands (0 h – no cell: stainings of the adhesive island before cell seeding) and left to adhere and spread for 24 h (24 h – with cell: stainings of the adhesive island after 24 h cell seeding). The substrates used were gold (Gold) and LBL-coated glass with macro-initiator printing (Macro I). For each series, the left image is an individual collagen I-FITC image and the right image is an overlay of at least 10 individual images.

Collagen I redistribution remained modest on both gold and macro-initiator patterned glass. Upon spreading on a patterned substrate, single cells exert forces on the ECM proteins or molecules (e.g. peptide sequences) to which they adhere. Therefore, providing these molecules are only loosely anchored to the underlying matrix or substrate, they can be detached, which results in their redistribution and the rounding up of cells [41]. In order to quantify collagen redistribution, [T]-shaped islands were patterned on both gold and glass. The [T] shape allows better observation of collagen redistribution as the cell body overlaps with uncoated areas. It was not possible to generate such islands on glass using silane **2** stamping, presumably due to the smaller size of the features ([T] consisted of two 10 μm-wide segments) for which lateral diffusion may be more detrimental. The [T] shapes are well suited for quantifying collagen redistribution because single cells that spread on these shapes present projected areas that overlap partially with the pattern and partially with the background (the non-adherent areas). Hence it is possible to visualise more easily the amount of collagen that is removed from the adherent areas by comparing heat maps of collagen staining before cell seeding and 24 h later (Fig. 7).

Analysis of heat maps indicated that HEKs seeded on patterned gold substrates do not remodel significantly the ECM adhesive island on which they spread and that their shape remains tightly controlled by the initial shape of each distinct pattern. Little collagen was imaged on the non-adherent areas of the projected cell body. HEKs seeded on glass substrates patterned with macro-initiator **3** redistributed collagen to a greater extent as evidenced by a more intense fluorescence signal in the central area of the pattern. However, these cells remained spread after 24 h seeding (Fig. 7) perhaps because cells still found sufficient amounts of collagen for spreading or possibly because they had replaced it with their own secreted matrix.

**Fig. 8:**
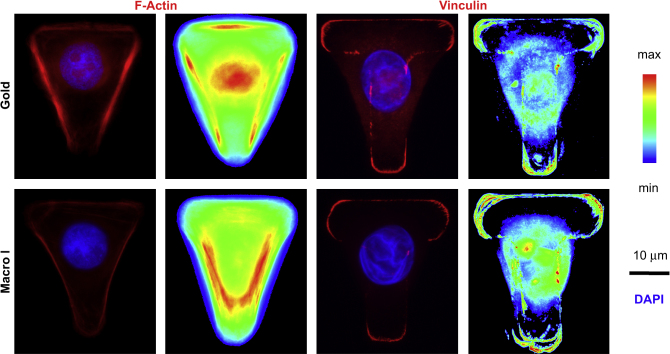
Cell polarisation induced with [T]-shaped adhesive islands. HEKs were seeded on [T]-shaped collagen I adhesive islands and left to spread for 3 h before fixation and staining for F-actin (red), vinculin (red) and DAPI (blue). The substrates used were gold (Gold) and LBL-coated glass with macro-initiator printing (Macro I). For each series, the left image is an individual staining image and the right image is an overlay of at least 15 individual images.

Finally, tight control over cell spreading resulted in the polarisation of cells, at least in terms of cytoskeletal organisation. Here, polarisation is defined as the lowering of the symmetry of cell structure (e.g. distribution of particular proteins or complexes). Several reports have shown that the sub-cellular organisation of cells [16] and the orientation of the mitotic spindle [14] are directed by the topology of the ECM on which cells spread. The effect of spreading of HEKs on [T] shapes was studied using phalloidin- and immuno-staining for F-actin networks and focal adhesion localisation (using primary antibodies raised against vinculin, Fig. 8). The heat maps obtained from the overlay of F-actin images (both on gold and glass patterns) clearly shows the formation of stress fibres along the non-adhesive edges of the cells, whereas focal adhesion markers were observed primarily at the three corners of the triangle formed by the adhering cell. The induction of such cytoskeletal polarisation enables cells to maximise their spreading, whilst maintaining a low membrane curvature, in very good agreement with the literature describing the behaviour of other cell types on ECM micro-patterns [42]. Hence, cell polarisation can be induced solely via the topology of the underlying ECM pattern, a phenomenon which may be useful for investigating the molecular mechanisms responsible for generating and maintaining cell polarisation without the use of chemical gradients or scrape wounding.

### 3.5 Live microscopy of patterned single cells

In some cases, cell patterning experiments require the imaging of cells live, as this is one of the only methods that can fully capture the inherent dynamic nature of cell biology. Therefore, it was important to assess whether our patterning technique, more specifically the type of substrates used, is compatible with state-of-the-art imaging technologies. For experiments in which cells are fixed and the substrates mounted on a second coverslip, imaging does not have to be carried out through the gold layer. However, many live imaging platforms require both excitation and emitted light to travel through the substrate.

The performance of four different types of substrates was examined by UV–vis absorption spectrophotometry as well as fluorescence and FRET imaging: non-coated glass and 2-, 4- and 15-nm gold-coated coverslips. The UV–vis absorption spectra of the three coated substrates display a gradual increase of the background level, although modest (Fig. S5). Slides coated with 2-nm of gold only display absorption levels ranging between two and three times those of un-coated slides: the absorption arising from the 2-nm gold coating itself is equivalent to that of only 1–2 glass slides. In addition, the absorption spectrum of the 2-nm gold-coated slides is relatively flat in the 350–800 nm range (which is the useful range for most live cell fluorescence imaging), except for a very broad low peak centred at 650 nm (the peak-to-trough height is less than 0.02 a.u., compared to the 0.057 a.u. of maximal variation in absorption measured for a non-coated glass slide taking air as a reference). This lack of feature compared to non-coated glass slides is especially important for quantification of FRET measurements as it decreases the differential absorption of light emitted at different wavelengths, a parameter that needs to be minimised when calculating FRET efficiencies from the quencher and donor intensities. The absorption spectrum of 4-nm gold-coated slides is very similar to that of 2-nm slides, except for a shift in the peak to 667 nm and a rise in the position of the background. This contrasts slightly with the spectrum obtained for 15-nm gold-coated slides, for which the long-wavelength peak is further shifted batochromically and displays a maximum variation in absorption of 0.084 a.u. Therefore, 15-nm gold-coated slides should be less suitable for quantitative FRET imaging.

**Fig. 9:**
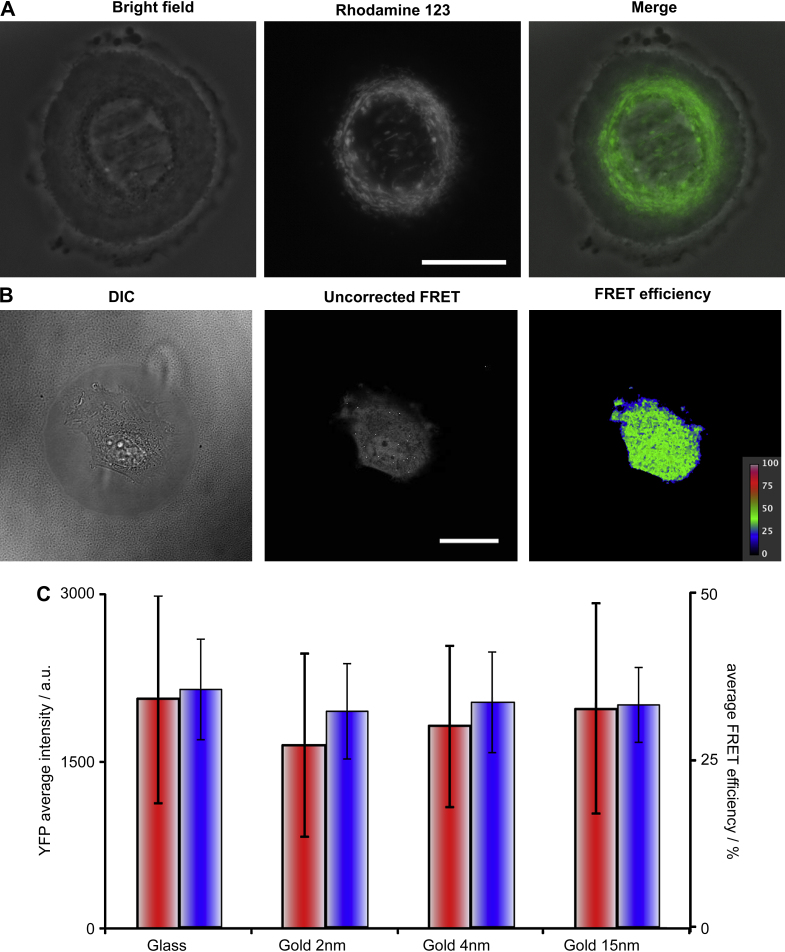
Live imaging of cells seeded on patterned substrates. (A) Live HEK cell spread on a 50 μm island (patterned on 15 nm gold) and labeled with rhodamine 123 (mitochondria staining) imaged by bright field and fluorescence microscopy. Scale bar: 20 μm. (B) NIH-3T3 fibroblast expressing a fusion protein of Cerulean and Venus linked by 5 aminoacids (C5 V) spread on 50 μm islands (patterned on 2 nm gold) and imaged by DIC and FRET microscopy. Scale bar: 20 μm. (C) Comparison of YFP fluorescence intensities (red bars) and corrected FRET efficiencies (blue bars) measured for at least 10 NIH-3T3 cells plated on different substrates. Mean ± SE; no statistical difference.

The quality of bright field and fluorescence microscopy images remains excellent on gold-coated substrates. Fig. 9A and B provide examples of fluorescence and FRET imaging carried out on live HEKs and fibroblasts seeded on 50 μm islands, stained for mitochondria (with rhodamine 123) and transfected with FRET probes, respectively. Although some intensity is lost, quantification can still be carried out providing that cells seeded on identical types of substrates are compared. In fact, when the total intensities of fibroblasts transfected with fluorescent probes and seeded on the different types of substrates are compared, no statistically significant difference can be measured (Fig. 9C). This suggests that although some light is absorbed by the gold coatings, this phenomenon is negligible compared to the fluorescence cell-to-cell variability, despite sorting transfected fibroblasts to retain a relatively narrow portion of cells expressing the transfected probe. Similar results were obtained for FRET efficiency measurements (Fig 9C). Hence the ultra-thin gold coatings used for our substrates are compatible with bright field, fluorescence and FRET imaging.

## 4 Conclusion

POEGMA brushes are particularly promising coatings for controlling protein adsorption at the micron scale and facilitate the patterning of single cells. Owing to their excellent anti-fouling performance and chemical stability upon storage or in tissue-culture conditions, their use and handling is simple: glass slides patterned with these coatings may be used for cell patterning and controlling the spreading of single cells without the requirement of complex patterning equipment or protocols, which makes these tools well suited to their use in biology laboratories. In addition, the possibility of generating cell polarisation simply via pattern topology may be useful for studying mechanisms underlying cell polarisation, but also responsible for maintaining sub-cellular organisation and architecture. Finally, the possibility of combining this cell patterning technique with high definition imaging technologies opens the door to a new generation of single cell studies: the behaviour of single cells (spreading, polarisation, differentiation, division) may be controlled using our micro-patterns and probed using a combination of live imaging and FRET.

## Appendix

Figures with essential colour discrimination. Most of the figures in this article have parts that are difficult to interpret in black and white. The full colour images can be found in the online version, at doi:10.1016/j.biomaterials.2010.02.066.
