# Supplementary material for: Exploiting the superior protein resistance of polymer brushes to control single cell adhesion and polarisation at the micron scale
Source: Biomaterials. 2010 Jun;31(18):5030–41. doi: 10.1016/j.biomaterials.2010.02.066 (PMC3712172; doi:10.1016/j.biomaterials.2010.02.066)
Supplement: Supplementary file 1 [file mmc1.pdf]

Exploiting the Superior Protein Resistance of Polymer Brushes to Control Single Cell Adhesion and Polarisation at the Micron Scale

Supplementary Information

Julien E. Gautrot,[1] *Britta Trappmann,*[1] *Fabian Oceguera-Yanez,*[2] *John Connelly,*[2] *Ximin He,*[1] *Fiona M. Watt,*[2] *Wilhelm T. S. Huck*[1,3]*

[1] Melville Laboratory for Polymer Synthesis, Department of Chemistry, University of Cambridge, Lensfield Road, Cambridge, CB2 1EW, UK.

[2] Wellcome Trust Centre for Stem Cell Research, University of Cambridge, Tennis Court Road, Cambridge, CB2 1RE, UK.

[3] Radboud University Nijmegen, Institute for Molecules and Materials, Heyendaalseweg 135, 6525 AJ Nijmegen, The Netherlands.

* To whom correspondence should be addressed E-mail: wtsh2@cam.ac.uk.

RECEIVED DATE (to be automatically inserted after your manuscript is accepted if required according to the journal that you are submitting your paper to)

SUPPLEMENTARY EXPERIMENTAL DETAILS

Kinetics of polymer brush growth. The kinetics of brush growth was monitored via ellipsometry (on dry films), using 1 cm^2 substrates cut in a silicon wafers (Compart Technology Ltd) coated with a thick gold film (15 nm chromium, 200 nm gold). A self-assembled monolayer (SAM) of thiol **1** was deposited onto these substrates by incubation in a 5 mM ethanolic solution overnight. For mixed monolayers, displaying reduced surface densities of initiator **1**, a mixture of **1** with octylthiol **5** (Aldrich) was used (the total concentration of thiols was kept constant at 5 mM).

Example of procedure for POEGMA-brush growth (other experimental conditions tested are gathered in FigS1, together with the obtained brush growth kinetics): a solution of $CuBr_2$ (9 mg, 40 μmol), bpy (160 mg, 1.0 mmol) and OEGMA (6.3 g, 17.5 mmol) in water (11 mL) was degassed using nitrogen bubbling for 30 min. CuCl (41 mg, 410 μmol) was added to this solution and the resulting mixture further degassed for 15 min before transferring to a flask containing the initiator-coated gold surface under inert atmosphere. The polymerisation was stopped at different time points, the resulting substrates washed thoroughly in deionised water and ethanol, dried in a stream of nitrogen and analysed by ellipsometry.

Stability of POEGMA brushes. Gold substrates (based on silicon wafers) coated with POEGMA brushes with various thicknesses (at full initiator density) and grafting densities (the initial thickness being kept between 13 and 23 nm) were immersed in a solution of 10 mL PBS, at room temperature. Their dry thickness was measured by ellipsometry at different time points. Before each measurement, the sample was taken out of the PBS solution, washed thoroughly with deionised water and dried in a stream of nitrogen. The PBS solutions were changed every two weeks.

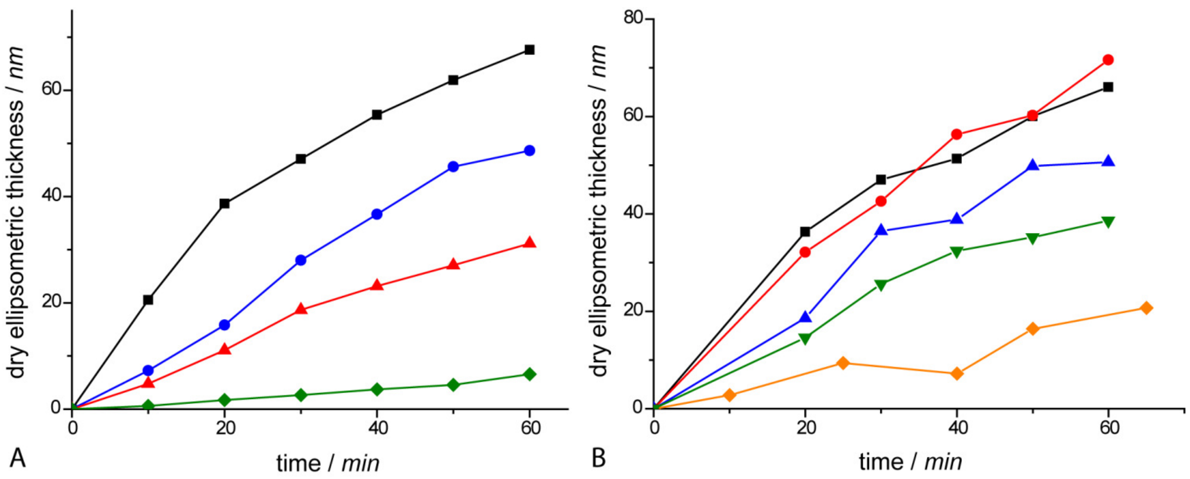

Fig. S1. Controlled growth of POEGMA brushes. A, Brush growth (dry ellipsometric thickness) from initiator **1** SAMs on gold, using: black squares, Cu(I)Cl (41 mg, 410 μmol) and Cu(II)Br_2 (9 mg, 40 μmol) in deionised water; blue circles, Cu(I)Cl (41 mg, 410 μmol) and Cu(II)Br_2 (18 mg, 80 μmol) in deionised water; red triangles, Cu(I)Cl (41 mg, 410 μmol) and Cu(II)Br_2 (9 mg, 40 μmol) in deionised water/methanol 8/3; green diamond, Cu(I)Cl (41 mg, 410 μmol) and Cu(II)Br_2 (9 mg, 40 μmol) in deionised water/methanol 5/6. B, Brush growth (dry ellipsometric thickness) from mixed initiator **1** and octylthiol **5** SAMs on gold, using Cu(I)Cl (41 mg, 410 μmol) and Cu(II)Br_2 (9 mg, 40 μmol) in deionised water; the SAMs were formed using ethanolic solutions containing initiator **1** at 100 (black squares), 45 (red circles), 31 (blue triangles), 14 (green inverted triangles), 4 (orange diamonds) mole%.

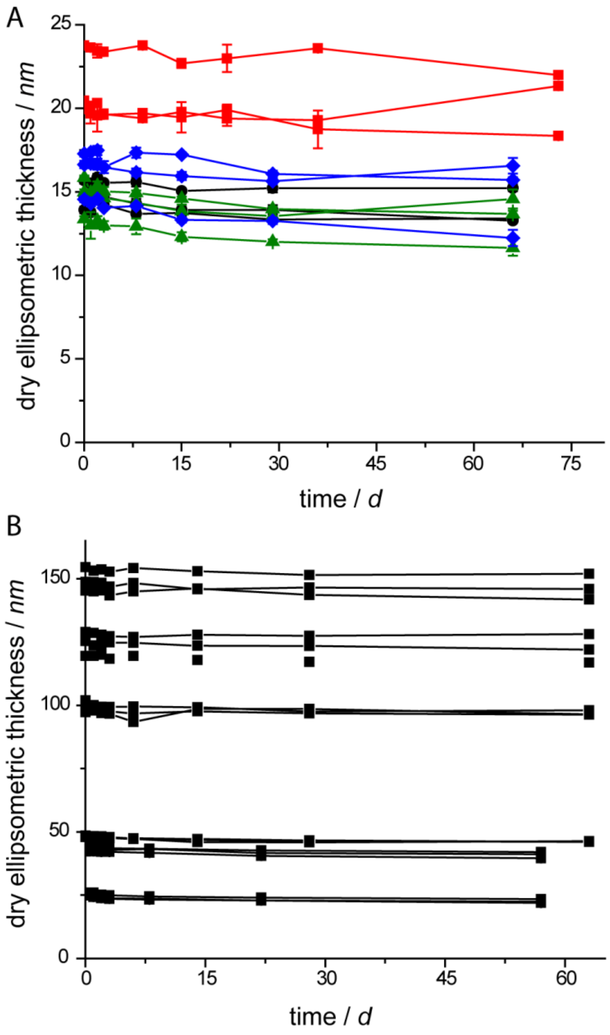

Fig. S2. Stability of POEGMA brushes in PBS. A, Decrease in brush dry thickness as a function of incubation time in a PBS solution, starting with brushes of varying grafting densities: brushes were grown on SAMs containing 45 (red squares). 31 (green triangles), 14 (blue diamonds), 4 (black circles) mole % of initiator **1**. B, Decrease in brush dry thickness as a function of incubation time in a PBS solution, starting with brushes of varying thicknesses (full grafting density). Measurements were carried out in triplicates. Incubations were performed at room temperature.

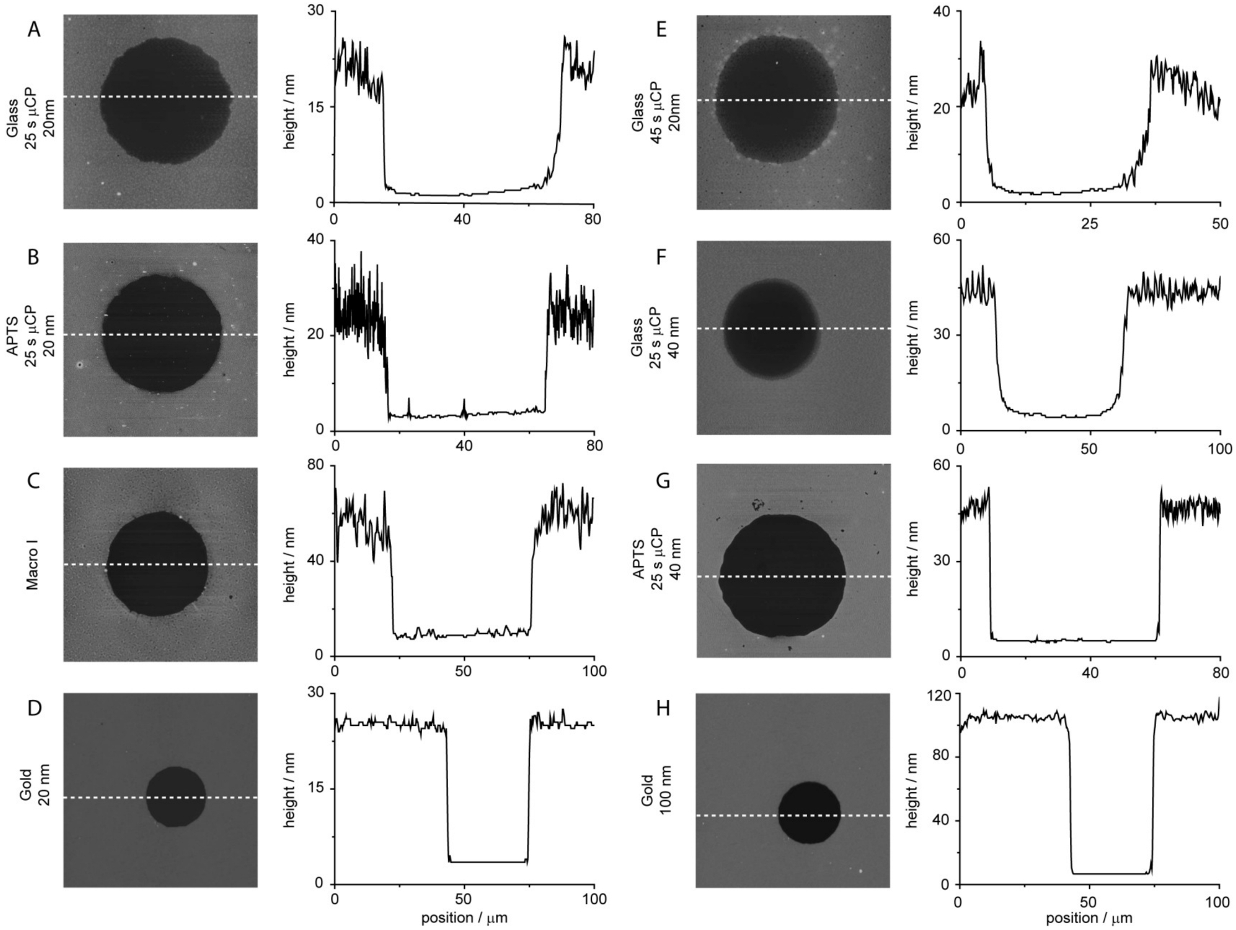

Fig. S3. Profiles of patterned POEGMA brushes analysed by AFM. A, glass (initiator **2** µCP for 25 s), B, APTS-coated glass (initiator **2** µCP for 25 s), C, LBL-coated glass (macro-initiator **3** µCP for 5 min), D, gold (initiator **1** µCP for 10 s), E, glass (initiator **2** µCP for 45 s), F, glass (initiator **2** µCP for 45 s, thick brush), G, APTS-coated glass (initiator **2** µCP for 25 s, thick brush), H, gold (initiator **1** µCP for 10 s, thick brush). The patterns in D, E and H are 30 µm circles (50 µm in all other images). Height profiles were measured along the dashed lines.

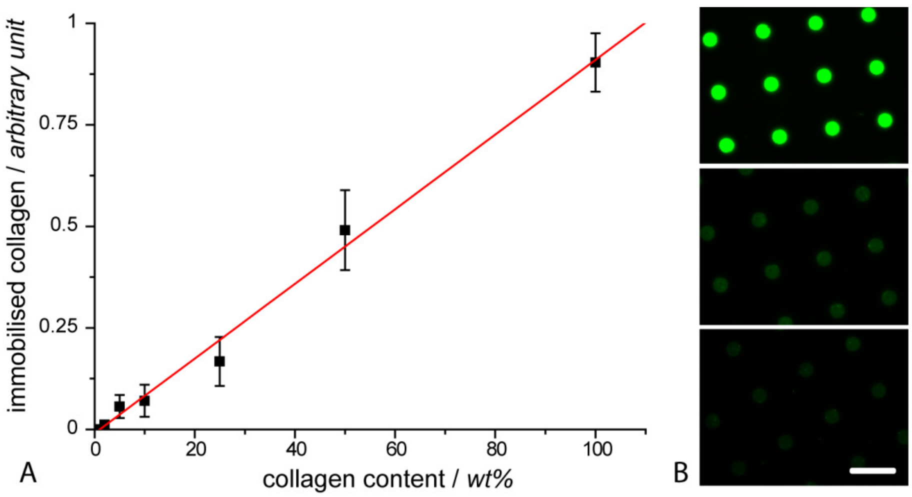

Fig. S4. Control of collagen I density on cell-adhesive islands. Substrates were incubated in collagen I and BSA mixed solutions. Collagen densities were measured by fluorescence imaging, after immuno-staining. Scale bar: 100 μm.

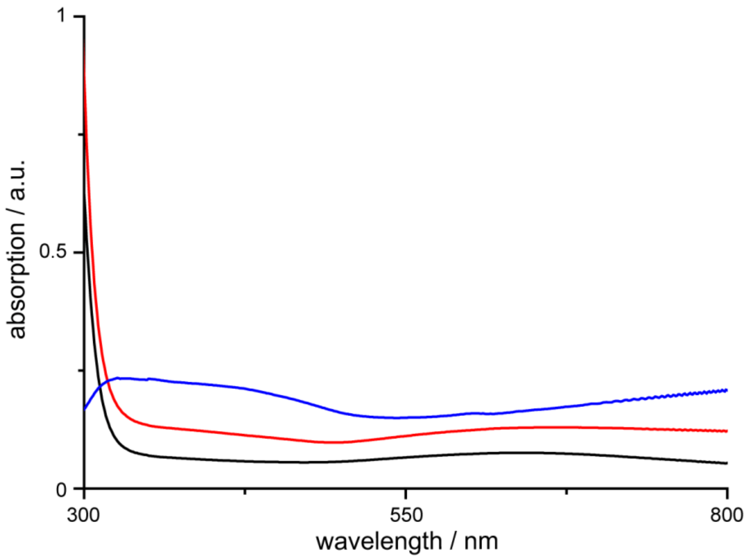

Fig. S5. UV-vis absorption spectrum recorded for 2 nm (black), 4 nm (red) and 15 nm (blue) gold-coated glass coverslips (a non-coated glass coverslip was used as reference).

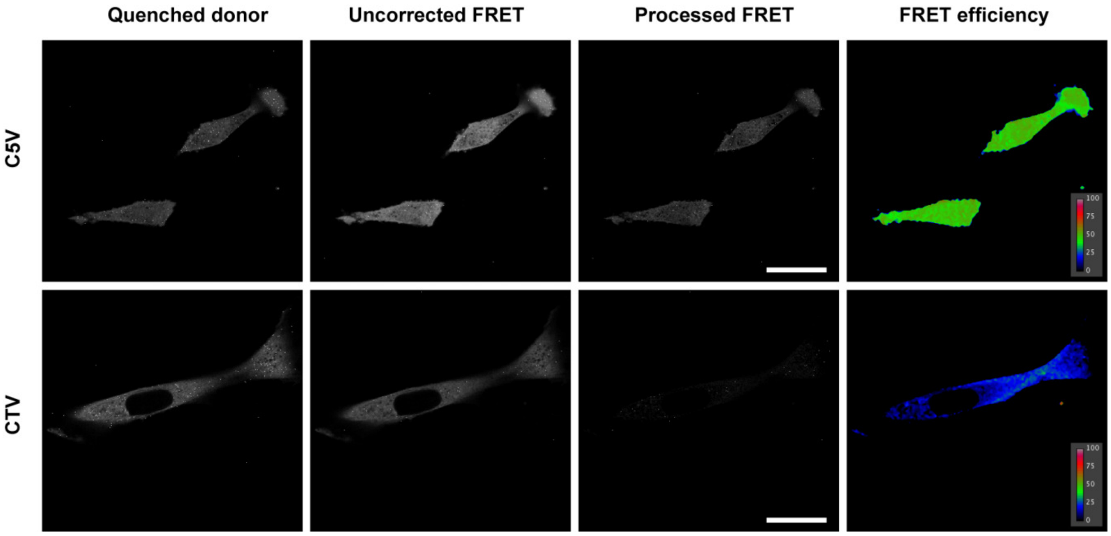

Fig. S6. Control FRET images for C5V (a construct encoding for Cerulean and Venus separated by 5 amino acids) used as a FRET positive control; or for CTV (Cerulean Venus construct separated by 229 amino acids) used as a FRET negative control. Transfected NIH-3T3 cells seeded on glass substrates (non-patterned) and imaged with a fluorescence confocal microscope. Scale bars: 20 μm.
